# A Crystalline 1D Dynamic Covalent Polymer

**DOI:** 10.1021/jacs.2c06446

**Published:** 2022-08-22

**Authors:** Elisabet De Bolòs, Marta Martínez-Abadía, Félix Hernández-Culebras, Alison Haymaker, Kyle Swain, Karol Strutyński, Benjamin L. Weare, Javier Castells-Gil, Natalia M. Padial, Carlos Martí-Gastaldo, Andrei N. Khlobystov, Akinori Saeki, Manuel Melle-Franco, Brent L. Nannenga, Aurelio Mateo-Alonso

**Affiliations:** † POLYMAT, University of the Basque Country UPV/EHU, Avenida de Tolosa 72, Donostia-San Sebastián 20018, Spain; ‡ Chemical Engineering, School for Engineering of Matter, Transport, and Energy, Arizona State University, Tempe, Arizona 85287, United States; § Center for Applied Structural Discovery, The Biodesign Institute, Arizona State University, Tempe, Arizona 85281, United States; ∥ CICECO - Aveiro Institute of Materials, Department of Chemistry, University of Aveiro, Aveiro 3810-193, Portugal; ⊥ School of Chemistry, University of Nottingham, University Park, Nottingham NG7 2RD, United Kingdom; # The Nanoscale and Microscale Research Centre, University of Nottingham, University Park, Nottingham NG7 2RD, United Kingdom; □ Instituto de Ciencia Molecular, Universidad de Valencia, Paterna 46980, Spain; ○ Department of Applied Chemistry, Graduate School of Engineering, Osaka University, Suita, Osaka 565-0871, Japan; △ Ikerbasque, Basque Foundation for Science, Bilbao 48009, Spain

## Abstract

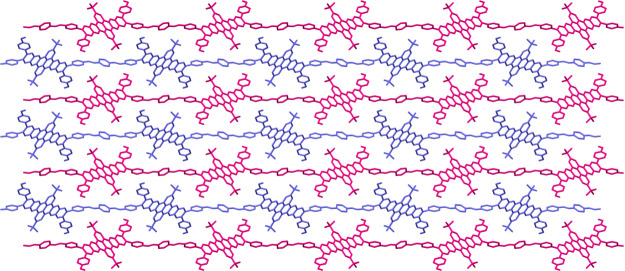

The synthesis of crystalline one-dimensional polymers provides a fundamental understanding about the structure–property relationship in polymeric materials and allows the preparation of materials with enhanced thermal, mechanical, and conducting properties. However, the synthesis of crystalline one-dimensional polymers remains a challenge because polymers tend to adopt amorphous or semicrystalline phases. Herein, we report the synthesis of a crystalline one-dimensional polymer in solution by dynamic covalent chemistry. The structure of the polymer has been unambiguously confirmed by microcrystal electron diffraction that together with charge transport studies and theoretical calculations show how the π-stacked chains of the polymer generate optimal channels for charge transport.

Synthetic polymers are essential materials in modern society. The thermal, mechanical, and conducting properties of polymeric materials depend directly on their degree of crystallinity. The preparation of crystalline one-dimensional (1D) polymers remains a challenge in chemistry because polymers tend to adopt amorphous or semicrystalline phases constituted of entangled polymer chains. The availability of crystalline polymers enables a fundamental understanding of their structure–property relationship but also opens the door for the preparation of new materials with enhanced properties.^1^ Topological polymerization has been used to obtain single crystals of 1D polymers.^2−10^ This is a crystal-to-crystal transformation in which monomers that have been preorganized in a crystal lattice undergo a solid-state polymerization reaction. The scope of topological polymerization is limited because the precursors must be crystallized and carefully arranged in the lattice for the polymerization to take place. The synthesis of crystalline 1D polymers in solution or in dispersed media has a much broader scope. However, the synthesis of crystalline 1D polymers in solution is quite challenging because of their high degree of conformational freedom in solution, which makes the organization of the polymer chains across the three dimensions of a crystalline solid difficult.

Dynamic covalent chemistry has shown great success in the solution synthesis of crystalline organic materials with different dimensionality, such as 1D,^11,12^ 2D,^13,14^ and 3D^13,15^ covalent organic frameworks. Under dynamic covalent chemistry conditions, the covalent bonds between the monomers can be formed and broken; hence, any structural error can be corrected by thermodynamic control. Approaches that combine dynamic covalent chemistry with templating motifs that limit the degrees of freedom of 1D polymers (e.g., coordination and hydrogen bonding) have been successfully used to weave,^16^ stiffen,^17,18^ and entwine^19^ polymer chains into crystalline superstructures. Yet, despite these impressive advances, the synthesis of disentangled crystalline 1D polymers from solution remains elusive.^1,18,20^

Herein, we report the synthesis of a disentangled and highly crystalline 1D dynamic covalent polymer (**Bet-P-1**, where Bet stands for Elisabet) in solution. **Bet-P-1** has been obtained by the substoichiometric (1:1) linkage of tetratopic and ditopic building blocks (respectively compounds **1** and **2**, Figure 1a) through reversible imine bonds. The crystal structure of **Bet-P-1** has been solved by microcrystal electron diffraction (MicroED) and shows that the disentangled polymer chains are tightly packed by π-stacking (Figure 1b–e). Electronic absorption and charge transport studies combined with theoretical investigations show that such interchain π-stacking opens up optimal channels for charge transport.

**Figure 1:**
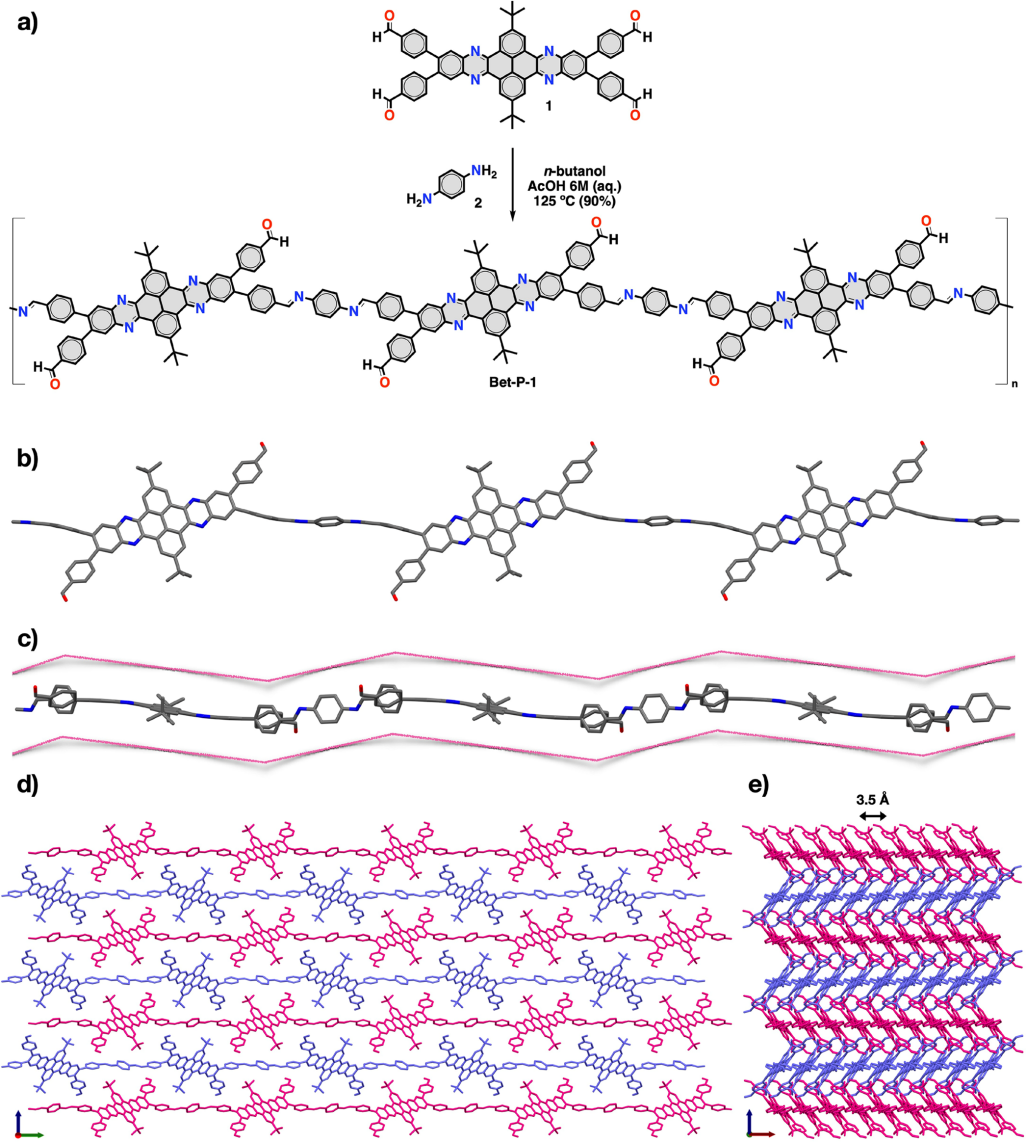
(a) Synthetic route for **Bet-P-1**. (b) Front and (c) side views of a single chain of **Bet-P-1** in the crystal structure. (d) Top and (e) side views of the crystal structure of **Bet-P-1** illustrating the packing of the different chains. Double arrow in the panel indicates the distance between the neighboring dibenzotetraazahexacene units. Hydrogens have been omitted for clarity.

We selected dibenzotetraazahexacene derivatives as nodes because of their synthetic accessibility, versatility, enhanced stability, and tendency to self-assemble by π-stacking.^21^ Dibenzotetraazahexacene **1** was synthesized in two steps from 2,7-di-*tert*-butyl-4,5,9,10-pyrenetetraone (**3**) (Scheme 1). First, condensation of pyrenetetraone **3**(22) with 2.1 equiv of 4,5-dibromo-1,2-phenylenediamine (**4**) in acetic acid yielded tetrabromodibenzotetraazahexacene **5** (95%) as a highly insoluble yellow solid that could be characterized only by ^1^H NMR in deuterated trifluoroacetic acid (TFA-*d*_1_) and MS. Then, Suzuki coupling between tetrabromodibenzotetraazahexacene **5** and 4-formylphenylboronic acid (**6**) yielded dibenzotetraazahexacene **1** (22%) as an orangish solid that shows an optimal solubility for solution synthesis.

**Scheme 1:**
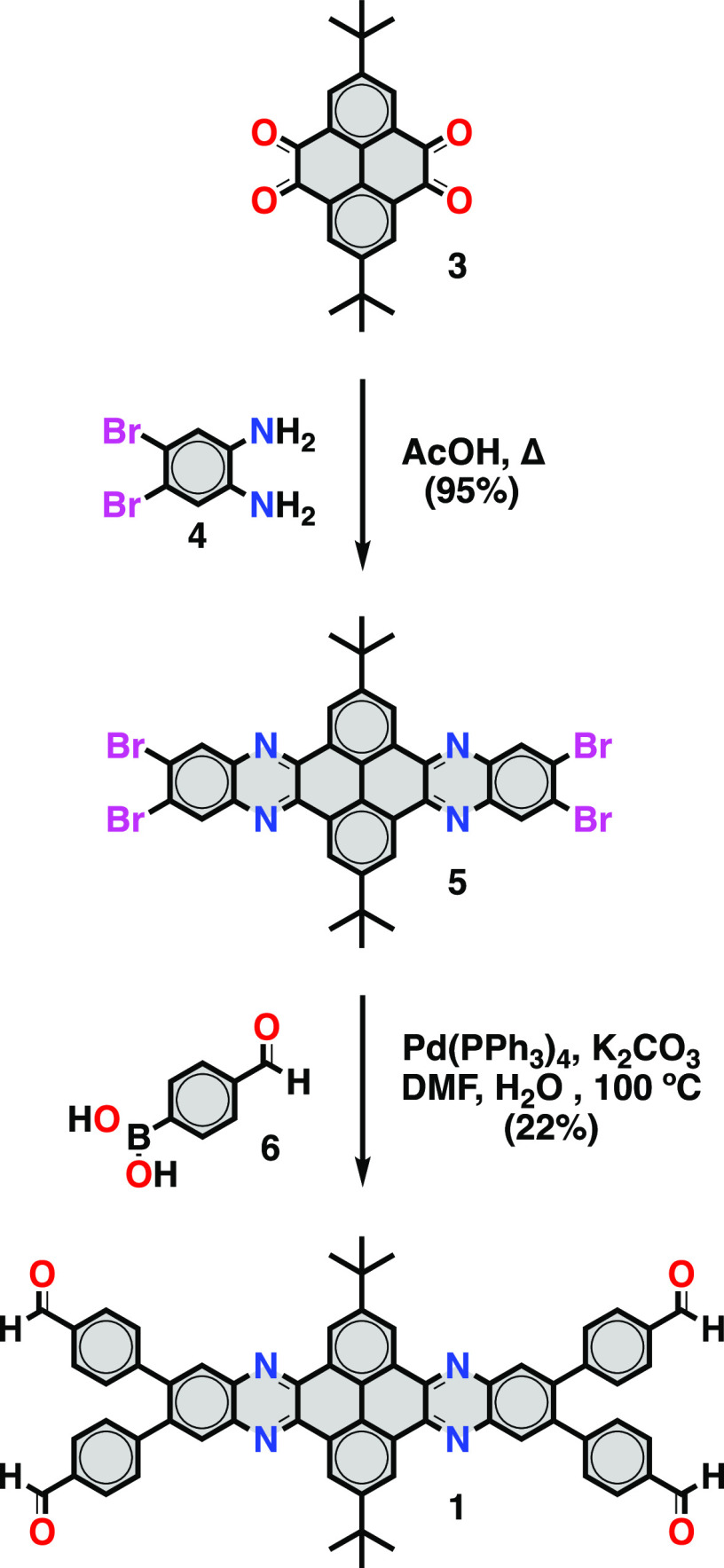
Synthesis of Precursor **1**

Initially, we screened several conditions for the condensation of dibenzotetraazahexacene **1** with *p*-phenylenediamine (**2**) in different solvent mixtures (*o*-dichlorobenzene, mesitylene, and *n*-butanol), different concentrations of aqueous acetic acid, and different stoichiometries (1:1, 1:2, 1:4). We used powder X-ray diffraction (PXRD) of the isolated solids to select the best conditions to obtain crystalline materials. Based on the screening experiments (a selection is shown in Figure S1), orangish crystalline powders of **Bet-P-1** were prepared in a 90% yield by condensation of a 1:2 molar mixture of dibenzotetraazahexacene **1** and *p*-phenylenediamine (**2**) in *n*-butanol in the presence of a 6 M aqueous solution of acetic acid (Figure 1a). Similarly to imine-linked covalent organic frameworks (COFs), **Bet-P-1** crystals show no sign of decomposition in nonprotic solvents such as acetone, chloroform, and hexane. Thermal gravimetric analysis under N_2_ shows that **Bet-P-1** is stable up to ∼500 °C (Figure S2).

The PXRD pattern of **Bet-P-1** suggests the formation of a highly crystalline material with a large number of sharp, well-resolved X-ray reflections (Figure 2a). Field emission scanning electron microscopy (FE-SEM) and high-resolution transmission electron microscopy (HR-TEM) show that the powders of **Bet-P-1** are constituted by needle-like microcrystals with the typical length of ca. 10 μm and diameters between 100 and 300 nm (Figures 2b, S3, and S4). FE-SEM imaging of surfaces of individual crystals indicated a helical twist of the crystal facets (Figure S3). High-magnification HR-TEM imaging of individual crystals reveals the presence of distinct lattice fringes that are separated by a distance of 1.4 nm running parallel to the main axis of the crystal (Figures 2c–e and S5 and S6). This further confirms the high crystallinity and also illustrates the dense packing of polymeric chains in **Bet-P-1**. This dense packing is also consistent with the nitrogen uptake measurements that show a virtually negligible adsorption and a very low Brunauer–Emmett–Teller (BET) surface area of 11 m^2^ g^–1^ (Figure 2f). For instance, the Zeo++ and Poreblazer surface areas calculated for the crystal structure of **Bet-P-1** are 0 m^2^ g^–1^ in both cases.

**Figure 2:**
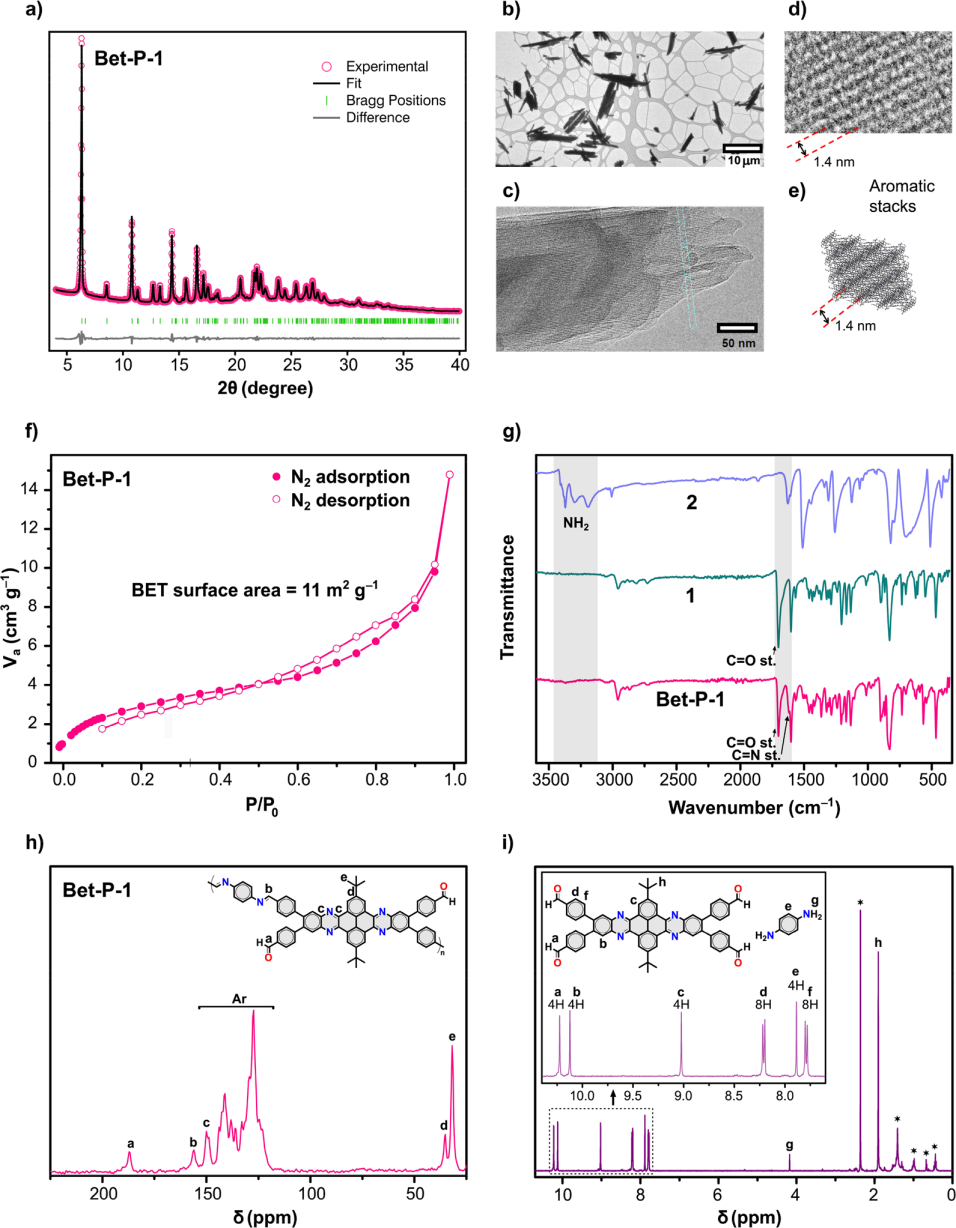
(a) Pawley refinement of the experimental diffraction data of **Bet-P-1** collected at room temperature. (b) Large field of view 200 kV TEM image of needle-like crystals dispersed on a lacey-carbon-coated grid. (c) HR-TEM image of an individual crystal revealing distinct lattice fringes running along the main axis of the crystal. (d, e) Separation of 1.4 nm between the areas of high contrast indicating aromatic moieties of the polymer forming (011) crystal planes. (f) N_2_ adsorption and desorption curves for **Bet-P-1**. (g) FT-IR spectra of *p*-phenylenediamine, **1**, and **Bet-P-1**. (h) CP/MAS ^13^C NMR spectrum of **Bet-P-1**. (i) ^1^H NMR spectrum of a hydrolyzed **Bet-P-1** sample in TFA-*d*_1_ showing the signals of dibenzotetraazahexacene **1** and *p*-phenylenediamine (**2**) monomers (*indicates solvent residual peak).

Although, theoretically, a 2D COF can be formed from a 1:2 molar ratio of dibenzotetraazahexacene **1** and *p*-phenylenediamine (**2**), the spectroscopic characterization shows that only two pairs of aldehydes were transformed into imines, generating a copolymer in 1:1 molar ratio. For instance, the Fourier transformed infrared (FT-IR) spectrum of **Bet-P-1** shows the imine C=N stretch band (1619 cm^–1^), whereas the aldehyde C=O stretch band (1701 cm^–1^) does not show any signs of attenuation (Figure 2g). This is in agreement with the cross-polarization/magic angle spinning (CP/MAS) solid-state ^13^C nuclear magnetic resonance (NMR) spectrum of **Bet-P-1**, which exhibited two signals that correspond respectively to the imine and to the unreacted aldehyde groups (Figure 2h). Additionally, the NMR spectrum of the hydrolysis/digestion of the **Bet-P-1** crystalline powders in TFA-*d*_1_ shows the signals of the hydrolyzed dibenzotetraazahexacene **1** and *p*-phenylenediamine (**2**) monomers in a precise 1:1 ratio (Figure 2i), in agreement with the 1:1 stoichiometry of the polymer, including some residual peaks of the solvents used during filtration such as acetone and hexane.

The crystal structure of **Bet-P-1** was solved by MicroED^23^ with data from four crystals at a specimen temperature of ∼100 K that were collected and merged together to produce a final refined structure at 0.80 Å with the *P*2_1_/*n* space group (Figure 1b–e; Table S1). The theoretical PXRD pattern of the crystal structure of **Bet-P-1** determined by MicroED shows an excellent correlation with the experimental PXRD pattern (Figure 2a). We used the Pawley refinement of the experimental PXRD pattern of the bulk material versus the monoclinic crystal structure of **Bet-P-1** determined by MicroED to confirm phase purity with excellent agreement factors (*a* = 6.18 Å, *b* = 16.36 Å, *c* = 26.58 Å, *R*_wp_= 2.39%, and *R*_p_= 1.67%) (Figure 2a; Table S2). The crystal structure unambiguously confirms that **Bet-P-1** is a linear polymer with a 1:1 molar ratio of monomers (Figure 1b,c), in which the dibenzotetraazahexacene nodes are covalently bound to the *p*-phenylenediamine linkers by two imines in a transoid configuration, leaving two unreacted aldehyde groups. **Bet-P-1** adopts a staircase-shaped conformation along the imine backbone (Figure 1c), where the 4-formylphenyl and the (4-formylphenyl)imino substituents are out of the plane of the dibenzotetraazahexacene nodes by 32° and 74°, respectively. The staircase-shaped linear polymer chains of **Bet-P-1** are π-stacked on top of each other at a distance of 3.5 Å (Figure 1e). These π-stacks crystallize in an antiparallel herringbone arrangement (Figure 1d), which gives rise to a densely packed crystal that is consistent with HR-TEM observations. For instance, the high contrast areas in the TEM micrographs correspond to the areas of highest density associated with the aromatic stacks of dibenzotetraazahexacene moieties, in particular in the (011) crystal planes (Figures 2c–e and S5 and S6). Based on the crystal structure of **Bet-P-1**, a potential rationale for the formation of a 1D polymer instead of a COF could be the excellent complementarity of the staircase-shaped linear polymer strands that act as polymeric docking sites, similarly to what was previously described for COFs obtained from stackable monomers.^24−27^

To shine some light on the optoelectronic properties of **Bet-P-1**, the solid-state UV–vis–NIR electronic absorption spectrum was compared with that of dibenzotetraazahexacene **1** (Figure 3a). The absorption spectrum of **Bet-P-1** shows absorption bands similar to that of dibenzotetraazahexacene **1** but slightly red-shifted, which is consistent with a more extensive π-stacking. The optical band gap of **Bet-P-1** estimated according to the Kubelka–Munk-transformed reflectance spectrum corresponds to 2.6 eV (inset Figure 3a).

**Figure 3:**
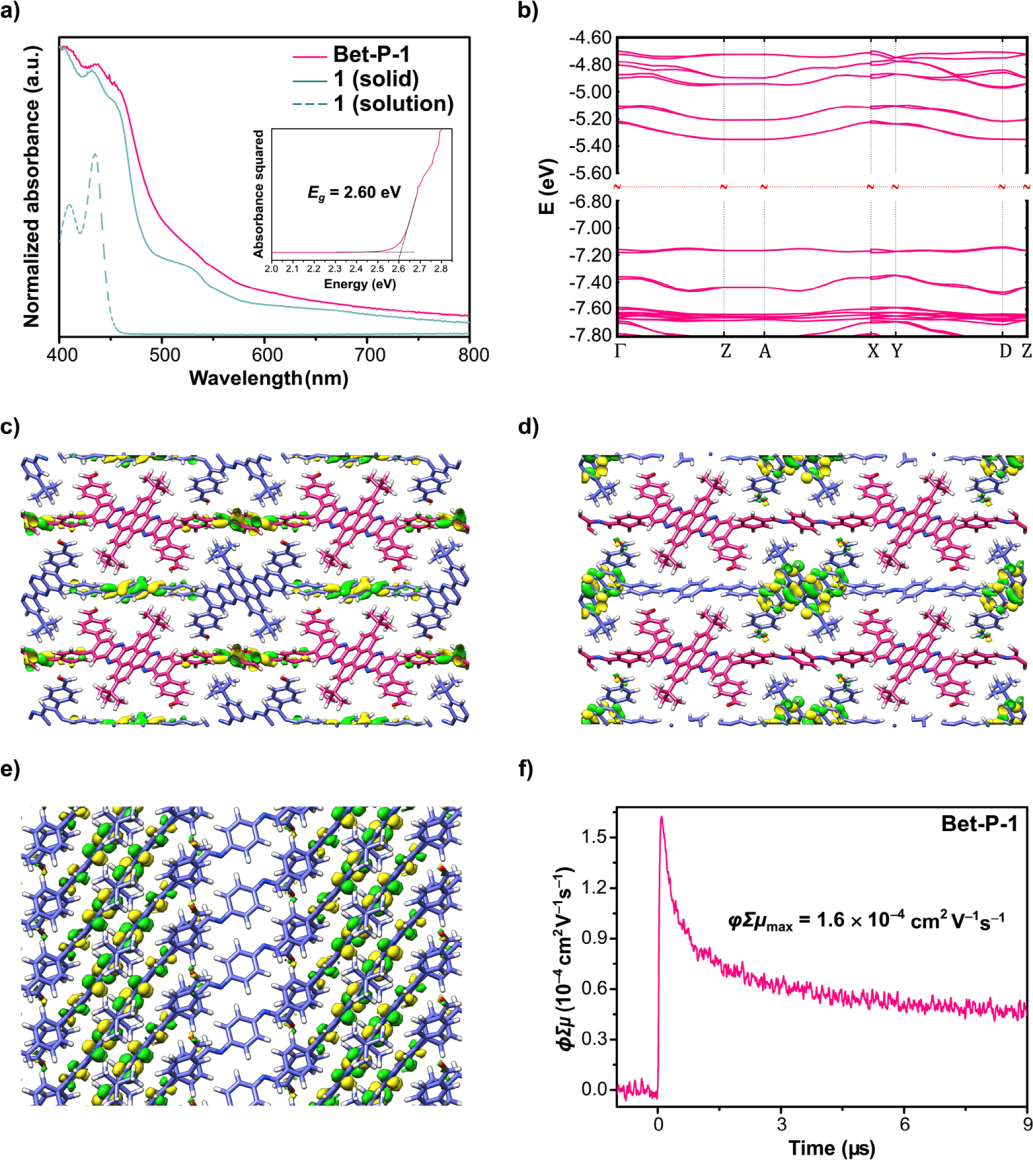
(a) UV–vis–NIR electronic absorption spectrum of **Bet-P-1**. The inset shows the estimated band gap. (b) Calculated band structure of the crystal structure of **Bet-P-1**. Top views of selected (c) HOCOs and (d) LUCOs of a **Bet-P-1** supercell. (e) Side view of the LUCO of a **Bet-P-1** supercell. (f) FP-TRMC conductivity transients of **Bet-P-1** upon excitation at 355 nm, 9.1 × 10^15^ photons cm^–2^ pulse^–1^.

To provide additional insights on the electronic structure and the charge-transporting properties of the microcrystals of **Bet-P-1**, we performed density functional theory (DFT) solid-state calculations with the PBE functional (details in the Supporting Information). The computed electronic band structure shows that **Bet-P-1** crystals are direct gap semiconductors with a band gap of 1.79 eV at the D-point (0.5 0.0 0.5) (Figure 3b). The highest occupied crystalline orbital (HOCO) band shows a weak bandwidth (40 meV), while the lowest unoccupied crystalline orbital (LUCO) band shows an increased bandwidth (120 meV), which can be mostly attributed to π–π stacking (see below). In addition, for increased accuracy, we also computed the band gap with the B3LYP hybrid functional, which yielded a value of 2.95 eV, similar to the band gap estimated experimentally. The frontier orbitals were analyzed in a 2×2×2 supercell computed in real space (details in the Supporting Information). In this system, the degenerate HOCOs show a localized electronic density on the phenylenebisphenylmethanimine residues (Figures 3c and S7), whereas the degenerate LUCOs show a localized electronic density on the dibenzotetraazahexacene nodes (Figures 3d and S7). Remarkably, due to the polymer intermolecular packing, the electron densities of some of the degenerate LUCOs spread throughout neighboring π-stacked dibenzotetraazahexacene units (Figure 3e), which opens up channels for electronic transport. To further confirm these channels, a detailed analysis of the bands was performed on a simplified unit cell representing a slab model of a 2D periodic system with an inclined unit cell sharing the same geometry of the bulk crystal but containing only one polymer strand parallel to the main unit cell axis with π–π stacking on the second unit cell axis (Figure S8 and Table S3). This model also shows that the most dispersive frontier band is the LUCO on the π-stacking direction with a bandwidth of 106 meV.

The charge transport properties of the crystalline powders of **Bet-P-1** were investigated by flash-photolysis time-resolved microwave conductivity (FP-TRMC).^28^ This technique measures the pseudophotoconductivity or the intrinsic charge carrier mobility (φΣμ, where φ is the quantum yield and Σμ is the sum of the charge carrier mobilities) directly on powders and films without the need of electrodes. FP-TRMC measurements on **Bet-P-1** show a maximum φΣμ value (φΣμ_max_) of 1.6 × 10^–4^ cm^2^ V^–1^ s^–1^ (Figure 3f). This φΣμ_max_ value is in the same range as the values observed on other π-stacked conducting systems, such as stacked pseudorotaxanes,^29^ supramolecular polymers,^30^ π-gels,^31,32^ covalent organic frameworks,^26,27,33−44^ and fused-aromatic networks,^45^ which show φΣμ_max_ values that oscillate between 10^–5^ and 10^–4^ cm^2^ V^–1^ s^–1^. The observed φΣμ_max_ value and the intermacromolecular spread of the electronic densities at the LUCO indicate that electronic transport takes place preferentially across the channels generated by the π-stacked dibenzotetraazahexacene residues of **Bet-P-1**.

To conclude, we have described the successful solvothermal synthesis of a disentangled crystalline 1D polymer (**Bet-P-1**) by dynamic covalent chemistry. The structure of **Bet-P-1** has been unambiguously confirmed by MicroED and is consistent with PXRD, HR-TEM, porosimetry, FT-IR, and NMR characterization. The crystal structure shows that the extended chains of **Bet-P-1** are π-stacked to one another through the dibenzohexacene units, which opens up channels optimal for charge transport. For instance, UV–vis–NIR electronic absorption, FP-TRMC, and theoretical calculations illustrate that **Bet-P-1** is a direct gap semiconductor with an intrinsic charge carrier mobility comparable to that observed in state-of-the-art π-stacked materials. This work illustrates that dynamic covalent chemistry is also a valuable tool for the synthesis of crystalline 1D polymers without the need of any templating strategy and paves the way for the synthesis of other families of crystalline 1D polymers with enhanced performance for electronic applications.

## Supporting Information Available

Experimental details of the synthesis, characterization, and calculations (PDF)
