# Supplementary material for: A Crystalline 1D Dynamic Covalent Polymer
Source: J Am Chem Soc. 2022 Aug 22;144(34):15443–50. doi: 10.1021/jacs.2c06446 (PMC9446889; doi:10.1021/jacs.2c06446)
Supplement: Supplementary file 1 — ja2c06446_si_001.pdf [file ja2c06446_si_001.pdf]

Supporting Information

A Crystalline 1D Dynamic Covalent Polymer

Elisabet De Bolòs,[1] Marta Martínez-Abadía,[1] Félix Hernández-Culebras,[1] Alison Haymaker,[2,3] Kyle Swain,[2,3] Karol Strutyński,[4] Benjamin L. Weare,[5] Javier Castells-Gil,[6] Natalia M. Padial,[6] Carlos Martí-Gastaldo,[6] Andrei N. Khlobystov,[5,7] Akinori Saeki,[8] Manuel Melle-Franco,[4] Brent L. Nannenga[2,3] and Aurelio Mateo-Alonso[1,9]*

[1]*POLYMAT, University of the Basque Country UPV/EHU, Avenida de Tolosa 72, Donostia-San Sebastián 20018, Spain. E-mail: amateo@polymat.eu*

[2]*Chemical Engineering, School for Engineering of Matter, Transport, and Energy, Arizona State University, Tempe, Arizona 85287, USA*

[3]*Center for Applied Structural Discovery, The Biodesign Institute, Arizona State University, Tempe, Arizona 85281, USA*

[4]*CICECO - Aveiro Institute of Materials, Department of Chemistry, University of Aveiro, Aveiro 3810-193, Portugal*

[5]*School of Chemistry, University of Nottingham, University Park, Nottingham NG7 2RD, United Kingdom*

[6]*Instituto de Ciencia Molecular, Universidad de Valencia, Paterna 46980, Spain*

[7]*The Nanoscale and Microscale Research Centre, University of Nottingham, University Park, Nottingham NG7 2RD, United Kingdom*

[8]*Department of Applied Chemistry, Graduate School of Engineering, Osaka University, Suita, Osaka 565-0871, Japan*

[9]*Ikerbasque, Basque Foundation for Science, Bilbao 48009, Spain*

Table of Contents

	Page
Supplementary Figures	S3
Supplementary Tables	S14
Experimental Procedures	S17
References	S26

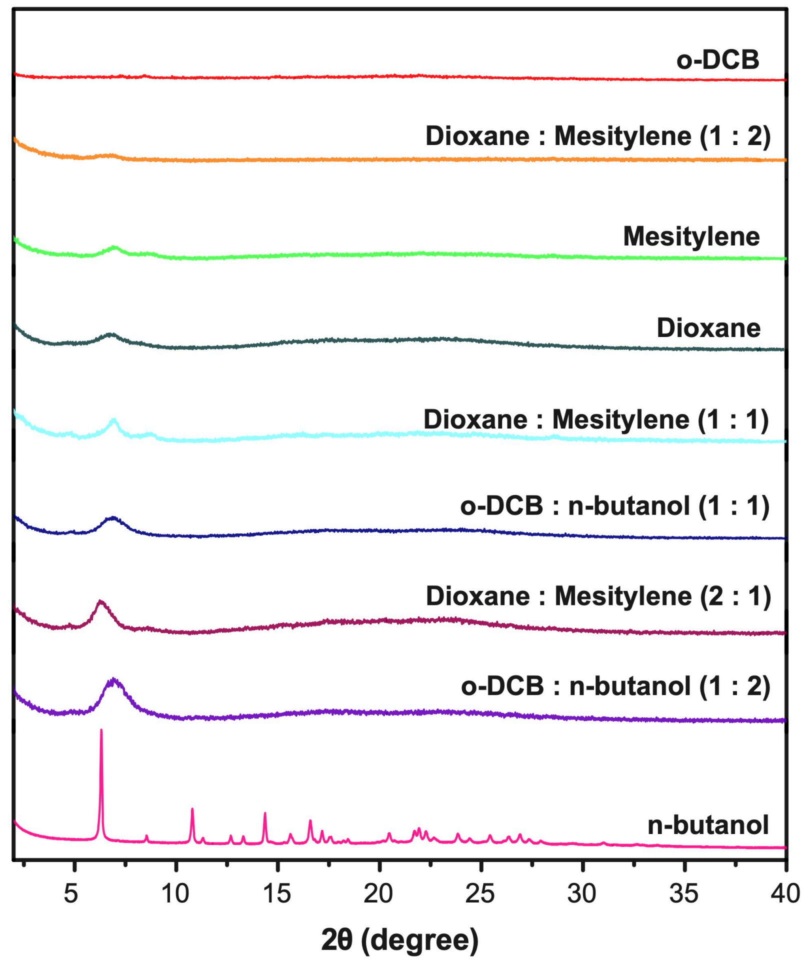

Figure S1. A selection of the solvents (indicated in the legend) used during the screening experiments and the corresponding PXRD patterns of the solids obtained (1:2 stoichiometry of building blocks **1** and **2**, 6M aqueous solution of acetic acid, 125 ºC, 5 days).

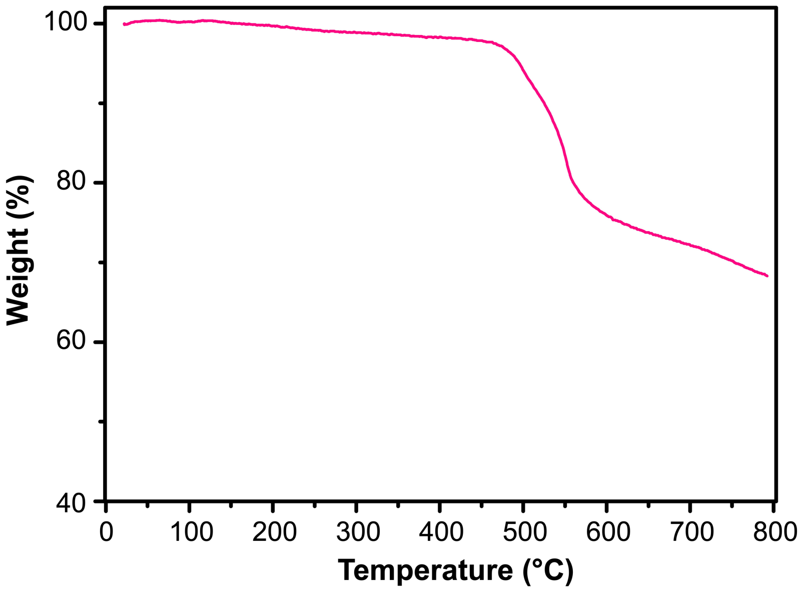

Figure S2. Thermal gravimetric analysis of **Bet-P-1** under N_2 (10 °C/min).

a)

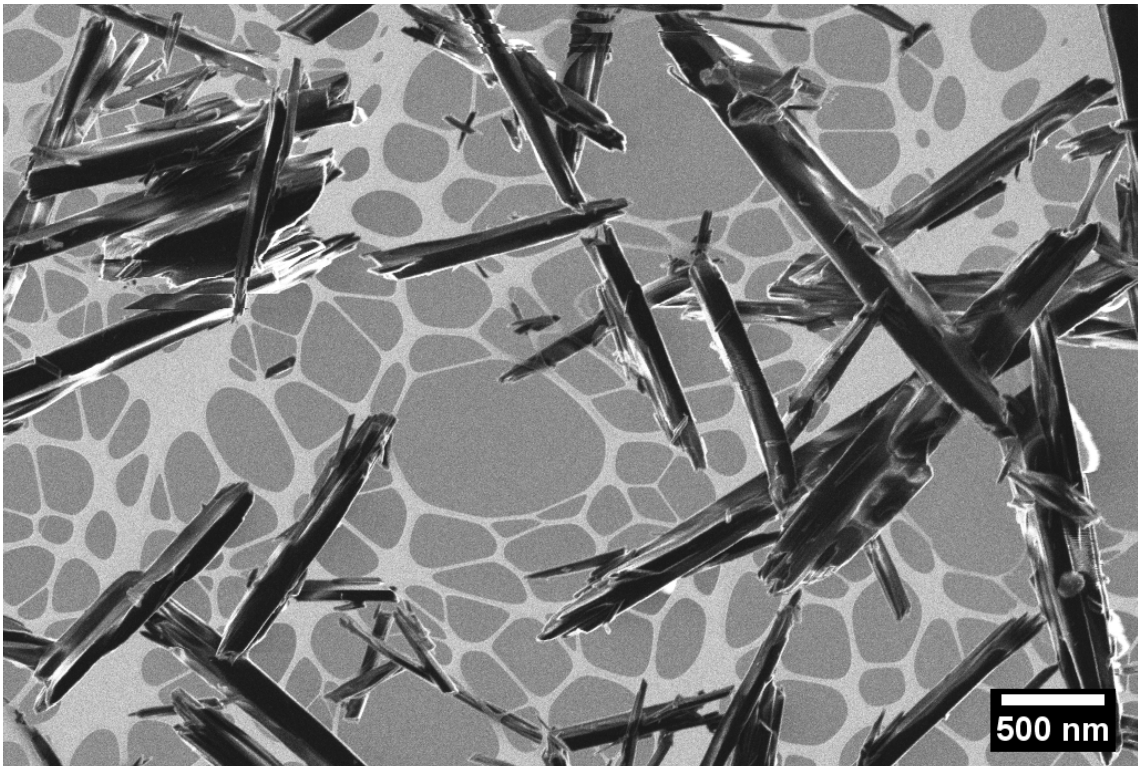

b)

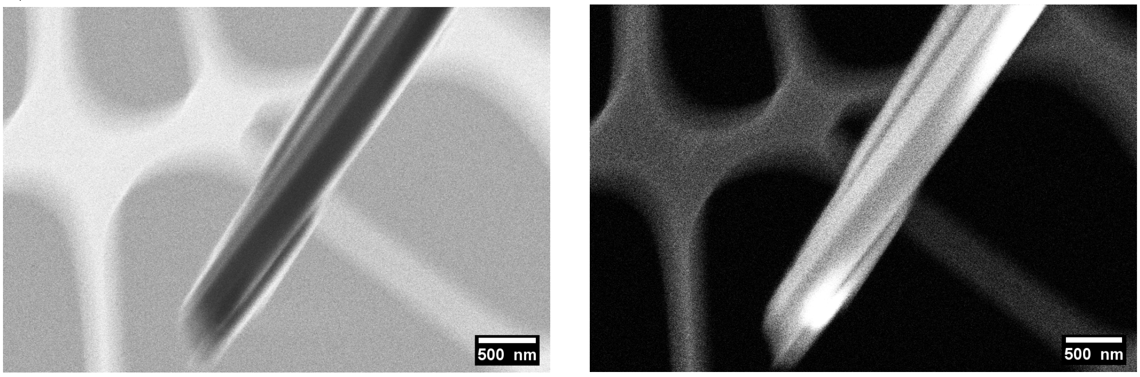

Figure S3. (a) SEM image of crystals of **Bet-P-1** dispersed on a lacey carbon TEM grid. (b) High magnification SEM images of individual crystal indicating a helical twist of the crystal faces.

a) b)

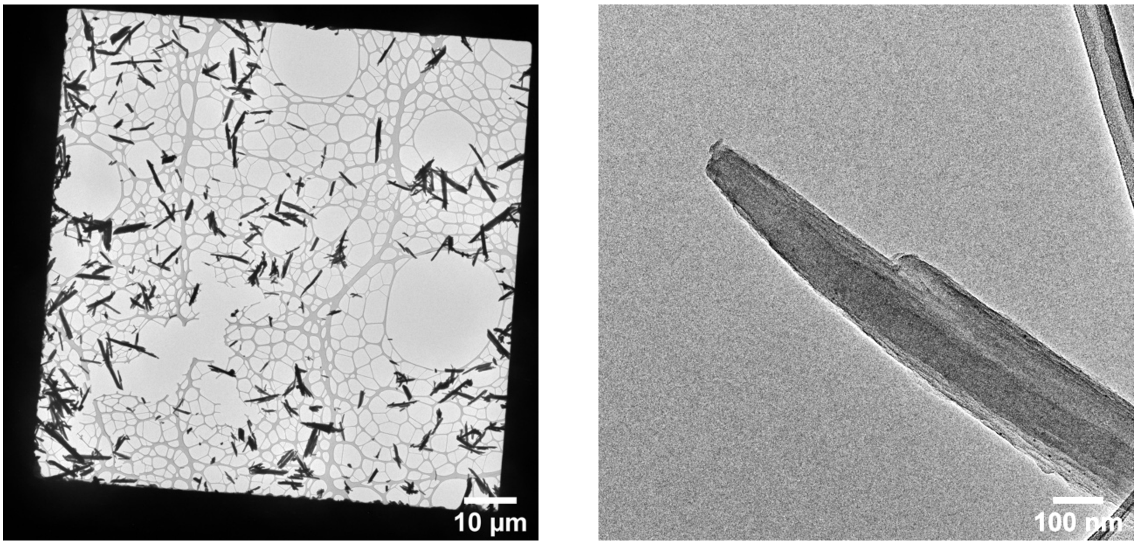

Figure S4. (a) Large field of view 200 kV TEM image of needle-like crystals of **Bet-P-1** dispersed on a lacey-carbon coated grid. images. (b) High-magnification TEM image of an individual crystal.

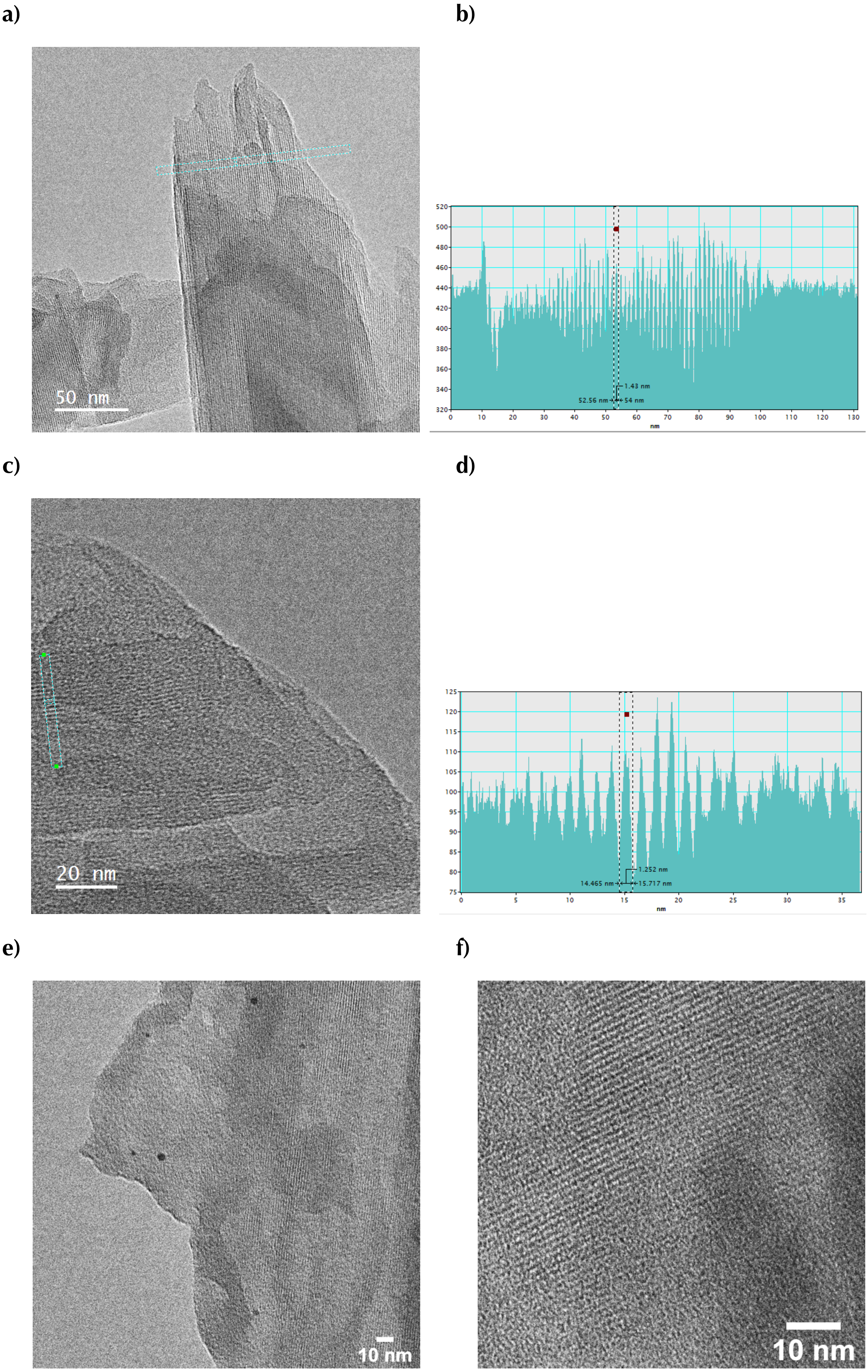

Figure S5. HRTEM image of a crystals of **Bet-P-1** recorded 200 kV (a, c). Line profile analysis showing planes with a periodic spacing of 1.3-1.4 nm (b, d). HRTEM image of **Bet-P-1** crystals recorded at 80 kV (e) and 200 kV (f).

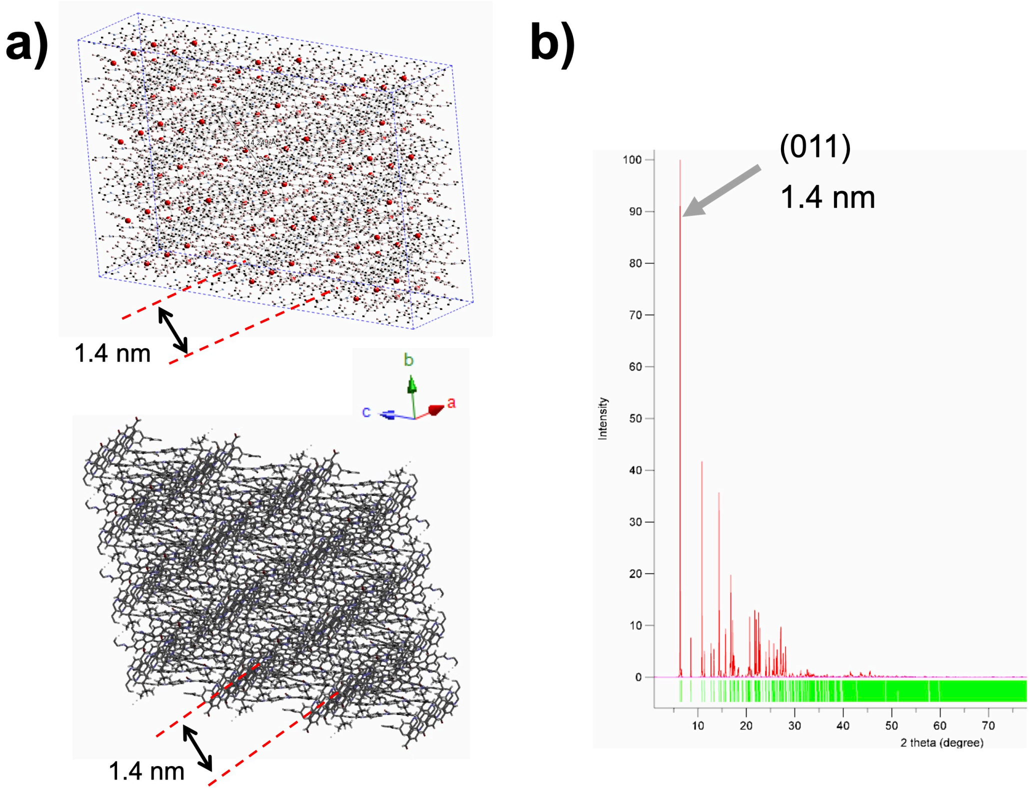

Figure S6. (a) Structural diagrams of **Bet-P-1** crystal lattice illustrating projections of (011) planes. (b) Simulated X-ray powder diffraction pattern from the atomic coordinates obtained from EBD data; (011) crystal planes correspond to the largest lattice spacing 1.4 nm that is observed in HRTEM images of **Bet-P-1** microcrystals (Figure 2d).

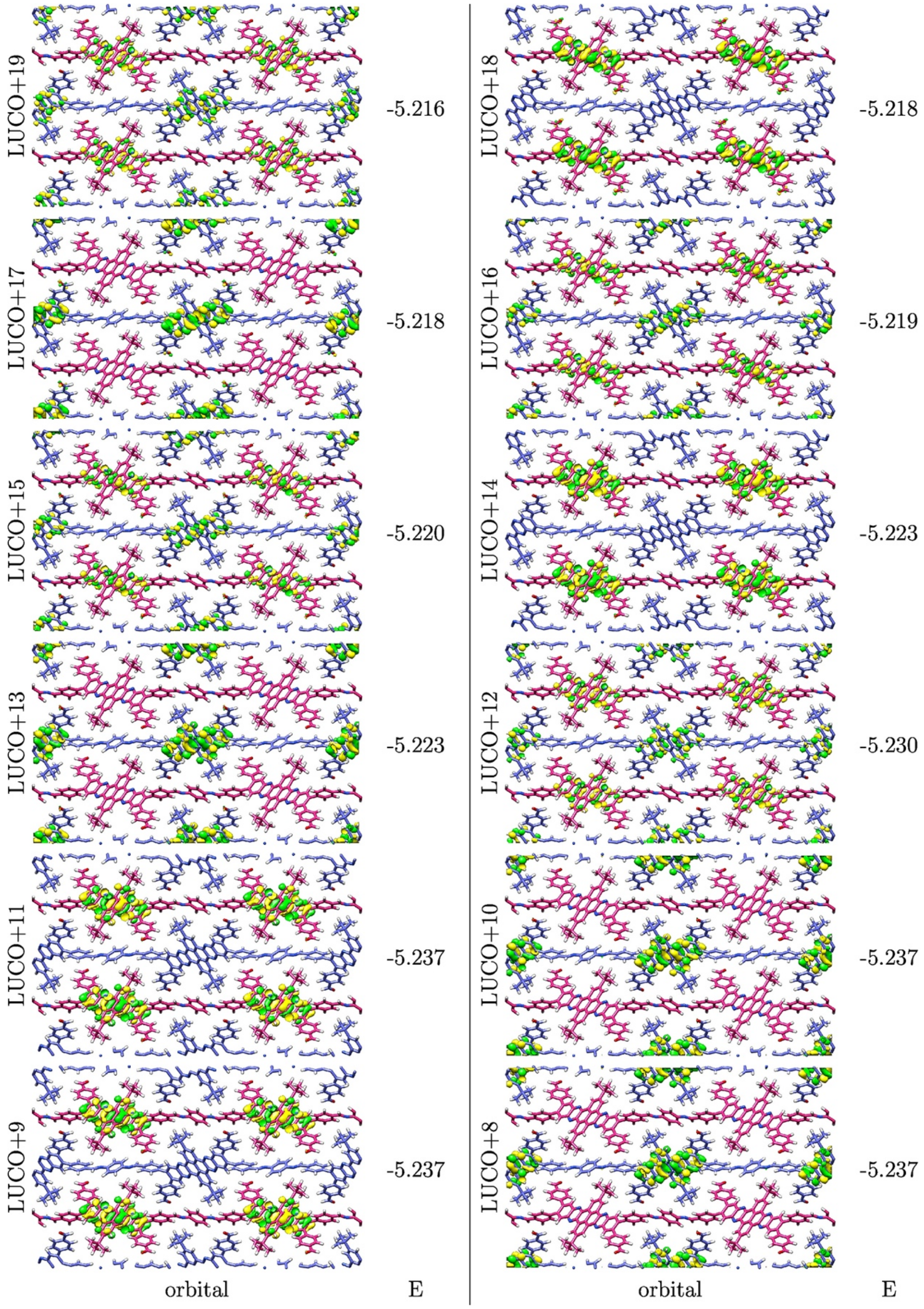

LUCO+19
-5.216
LUCO+17
-5.218
LUCO+15
-5.220
LUCO+13
-5.223
LUCO+11
-5.237
LUCO+9
-5.237
orbital
E
LUCO+18
-5.218
LUCO+16
-5.219
LUCO+14
-5.223
LUCO+12
-5.230
LUCO+10
-5.237
LUCO+8
-5.237
orbital
E

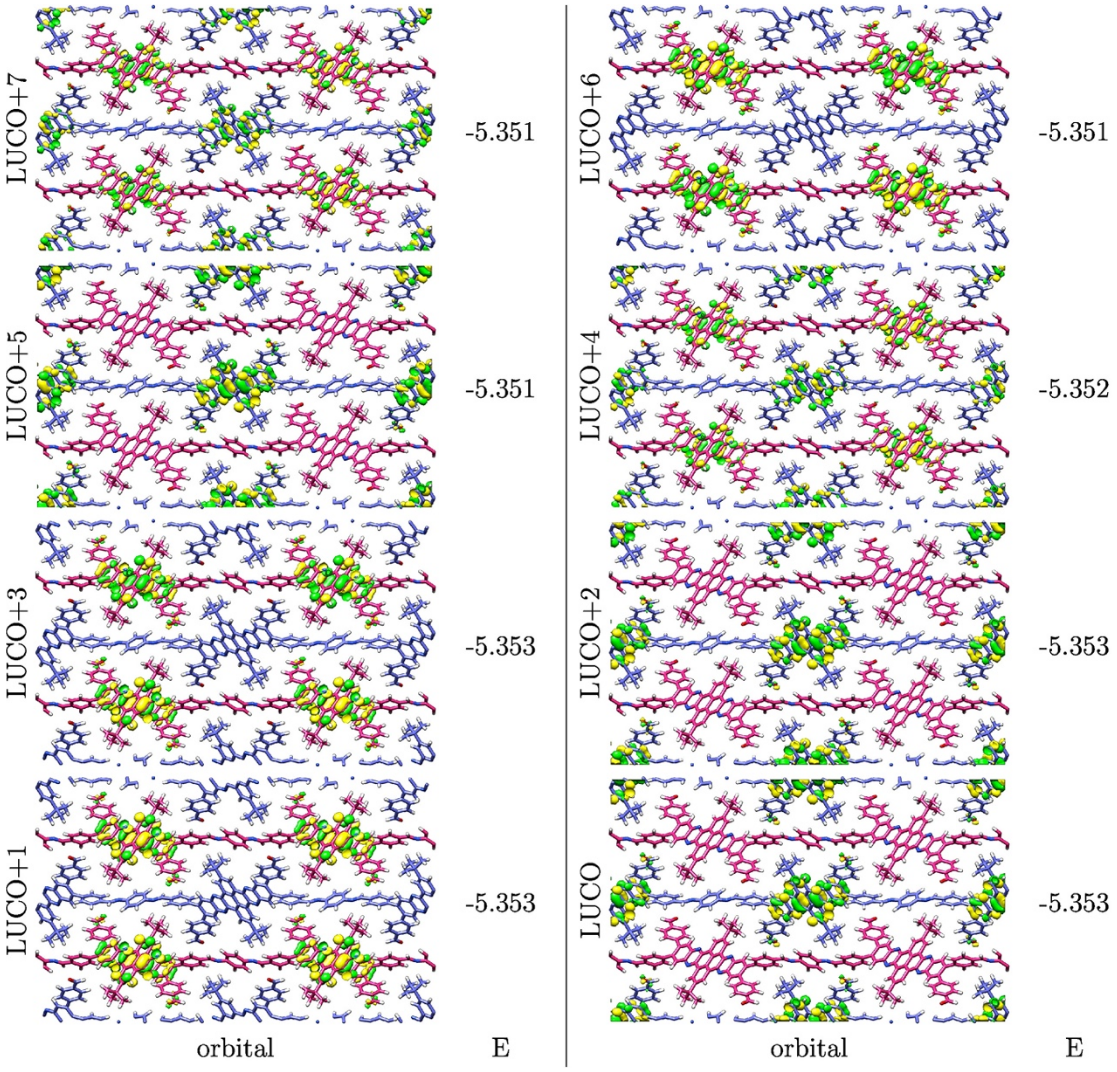
LUCO+7
-5.351
LUCO+6
-5.351
LUCO+5
-5.351
LUCO+4
-5.352
LUCO+3
-5.353
LUCO+2
-5.353
LUCO+1
-5.353
LUCO
-5.353
orbital
E
orbital
E

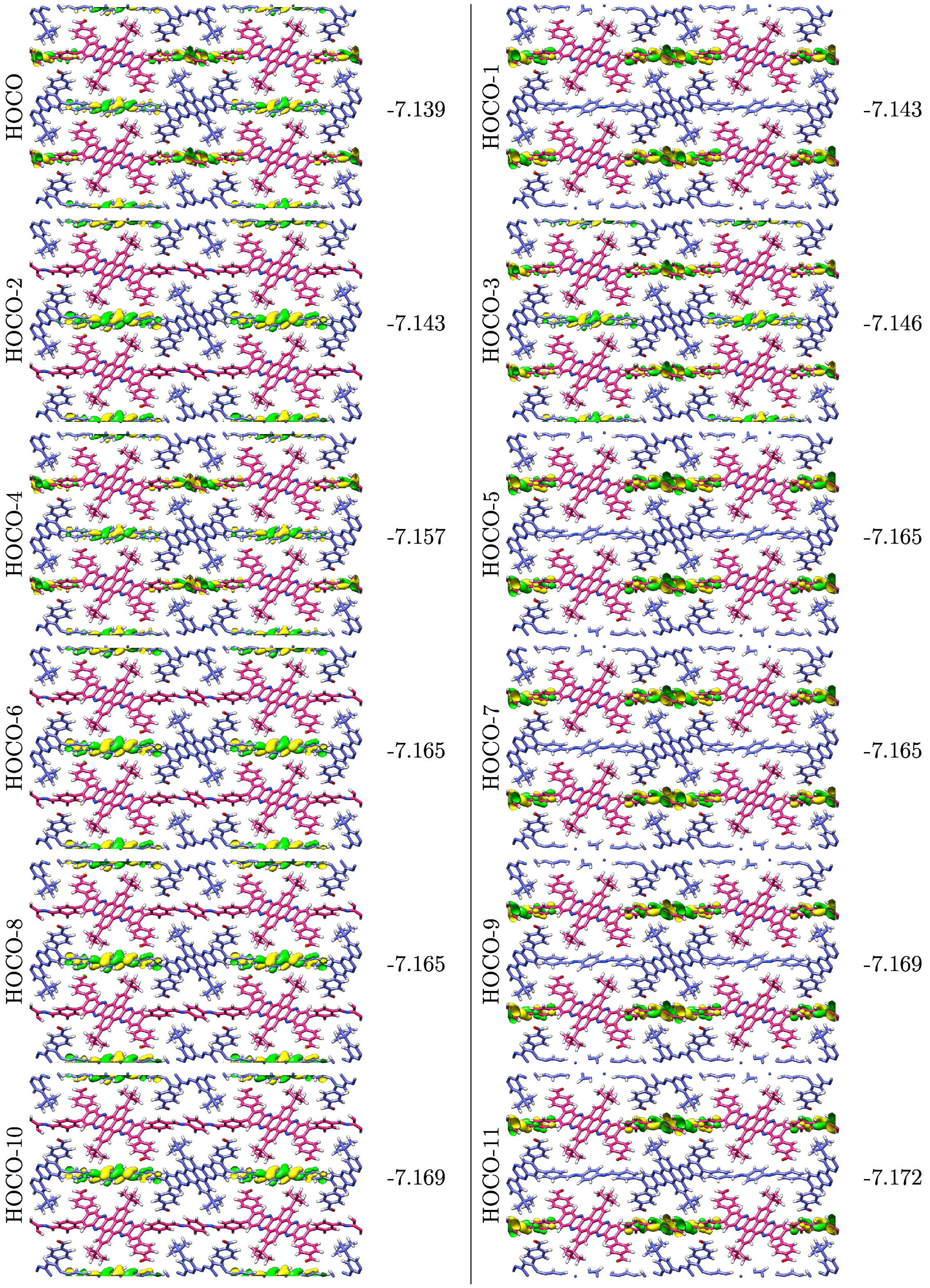
HOCO
-7.139
HOCO-1
-7.143
HOCO-2
-7.143
HOCO-3
-7.146
HOCO-4
-7.157
HOCO-5
-7.165
HOCO-6
-7.165
HOCO-7
-7.165
HOCO-8
-7.165
HOCO-9
-7.169
HOCO-10
-7.169
HOCO-11
-7.172

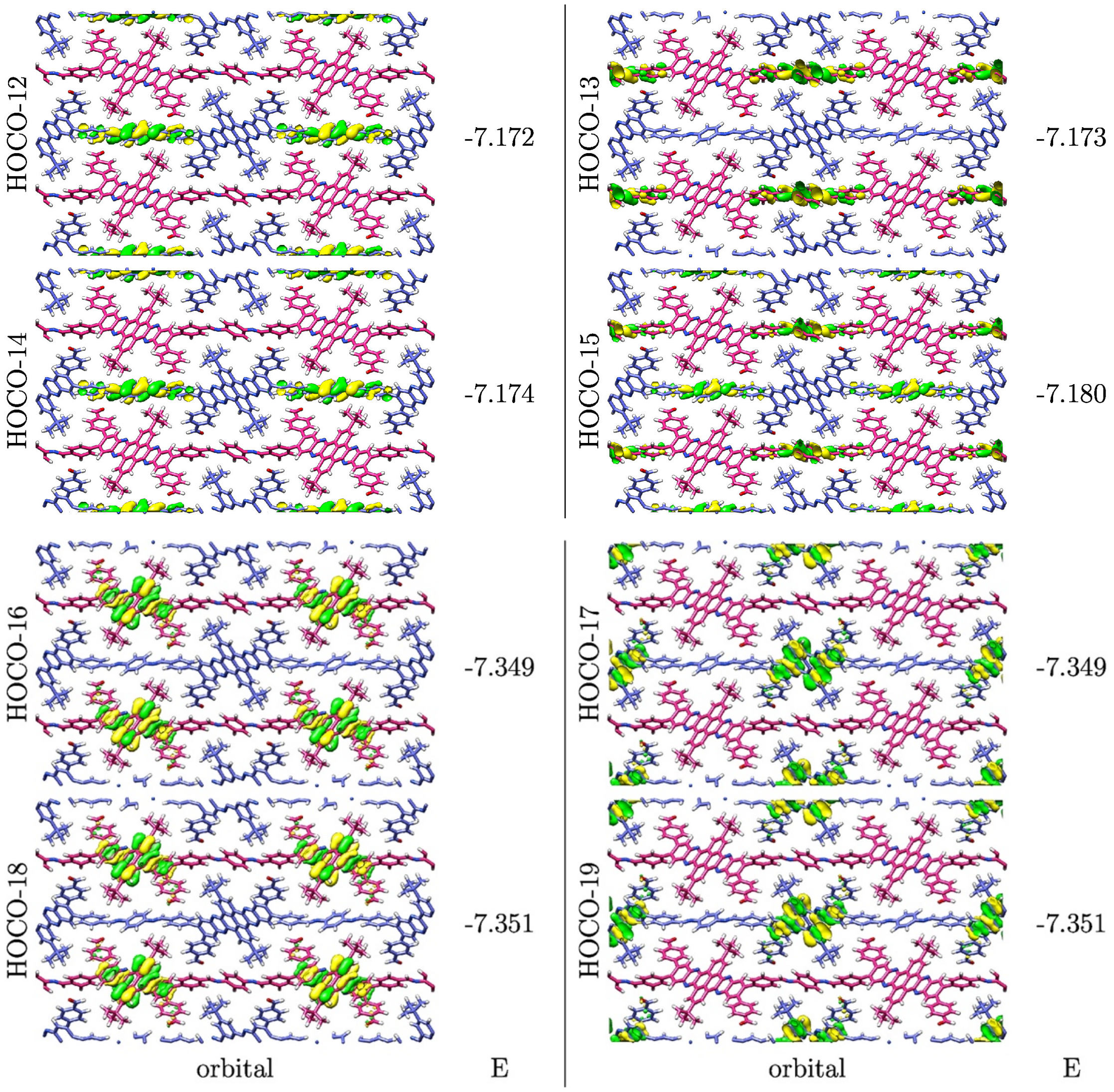

Figure S7. Selected molecular orbitals of **Bet-P-1** in bulk rendered in real space from **Γ**-point calculations on a 2x2x2 supercell. All energies are in eV.

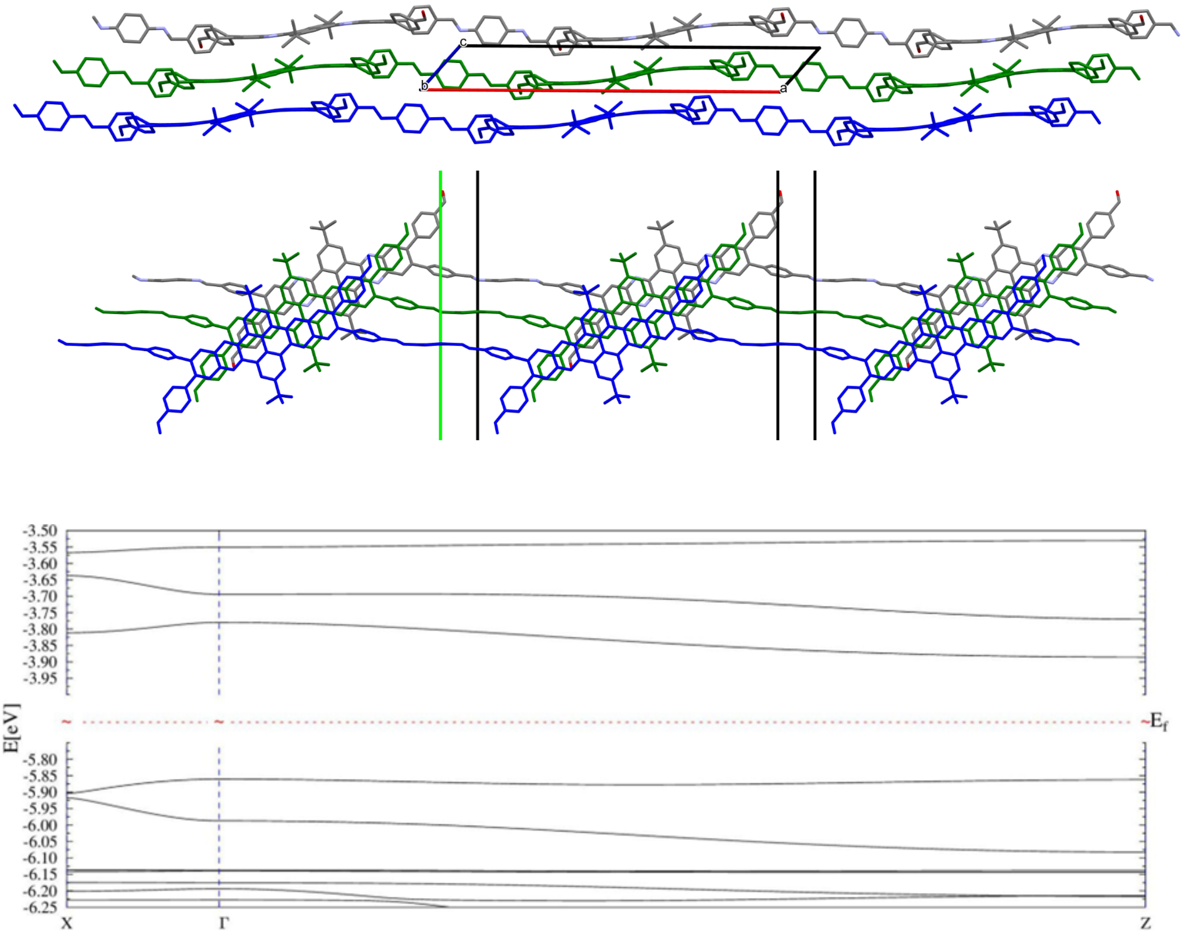

Figure S8. Two views of the simplified unit cell (top) and corresponding band structure on the intramolecular direction (bottom left) and intermolecular, π–π stacking, direction (bottom right).

Table S1. MicroED Data collection, processing and refinement statistics.

**Data collection**	
Excitation Voltage	300 kV
Wavelength (Å)	0.019687
Number of crystals	4
**Data Processing**[a]	
Space group	$P2_1/n$
Unit cell length a, b, c (Å)	6.17, 16.33, 26.63
Angles α, β, γ (°)	90.00, 90.49, 90.00
Resolution (Å)	10.00 - 0.80 (0.82 - 0.80)
Number of reflections	31,453
Unique reflections	4,831
R_{obs} (%)	20.2 (83.6)
R_{meas} (%)	21.9 (98.3)
I/σ_I	4.96 (0.80)
$CC_{1/2}$ (%)	98.4 (57.8)
Completeness (%)	85.0 (65.1)
**Structure Refinement**	
R1	0.2419
wR2	0.5777
GooF	1.962

[a] Values in parentheses represent the highest resolution shell

Table S2. Summary of the parameters obtained from the Pawley refinement.

Parameters	Refined Values
**a [Å]**	6.1795(5)
**b [Å]**	16.3572(8)
**c [Å]**	26.5853(9)
**β [°]**	88.997(7)
**V [Å^3]**	2686.8(3)
**R_e [%]**	0.92
**R_p [%]**	1.67
**R_{wp} [%]**	2.39
**GoF**	2.58

Table S3. Simplified unit cell bands maxima, minima and bandwidths.

		Intramolecular direction			Intermolecular (stacking) direction		
Cristalline Orbitals	band #	minima / eV	maxima / eV	bandwidth / meV	minima / eV	maxima / eV	bandwidth / meV
LUCO+9	274	-2.02	-2.01	9	-2.09	-2.02	71
LUCO+8	273	-2.05	-2.04	6	-2.13	-2.04	88
LUCO+7	272	-2.45	-2.43	20	-2.43	-2.30	134
LUCO+6	271	-2.87	-2.85	16	-2.87	-2.77	91
LUCO+5	270	-2.93	-2.93	2	-2.93	-2.82	108
LUCO+4	269	-3.09	-3.07	21	-3.18	-3.09	90
LUCO+3	268	-3.18	-3.14	36	-3.26	-3.14	119
LUCO+2	267	-3.48	-3.47	16	-3.47	-3.45	20
LUCO+1	266	-3.61	-3.55	57	-3.68	-3.61	77
LUCO	265	-3.73	-3.69	32	-3.80	-3.69	**106**
HOCO	264	-5.82	-5.78	43	-5.79	-5.78	17
HOCO-1	263	-5.90	-5.83	70	-6.00	-5.90	95
HOCO-2	262	-6.05	-6.05	1	-6.05	-6.05	2
HOCO-3	261	-6.06	-6.05	2	-6.06	-6.05	4
HOCO-4	260	-6.09	-6.09	1	-6.13	-6.09	40
HOCO-5	259	-6.12	-6.11	8	-6.15	-6.11	37
HOCO-6	258	-6.14	-6.14	1	-6.30	-6.14	156
HOCO-7	257	-6.28	-6.27	3	-6.41	-6.24	164
HOCO-8	256	-6.32	-6.28	42	-6.43	-6.28	153
HOCO-9	255	-6.42	-6.37	47	-6.43	-6.37	58

Experimental procedures

Reagents for synthesis were, if not otherwise specified, purchased from Aldrich, TCI or Acros Organics. Column chromatography was carried out using Silica gel 60 (40-60 μm) from Scharlab. Analytical thin layer chromatography (TLC) was done using aluminum sheets (20x20 cm) pre-coated with silica gel from Merck. UV-active compounds were detected with a UV-lamp from CAMAG at wavelength λ = 254 or 365 nm.

2,7-Di-tert-butylpyrene-4,5,9,10-tetraone (**3**) was synthesized from pyrene following previous reported procedures.[1,2]

Bet-P-1 was synthesized in a pre-scored 2 mL ampoule from Aldrich. For its synthesis and purification anhydrous n-butanol (99.8%), anhydrous chloroform (99.8 %), anhydrous acetone (99.8%), and anhydrous hexane (97%) were purchased from Acros Organics. THF was dried using an Innovative Pure Solve solvent purification system.

NMR spectra in solution were recorded on Bruker Avance 400 MHz pulsed Fourier transform NMR spectrometer at room temperature using partially deuterated solvents as internal standards. Chemical shifts (δ) are denoted in ppm. Multiplicities are denoted as follows: s = singlet, d = doublet, t = triplet, m = multiplet, br = broad.

Solid-State ^{1}H and ^{13}C CP/MAS NMR spectra were recorded on a Bruker Avance III 400 MHz NMR spectrometer at a MAS rate of 12 kHz and a CP contact time of 2 ms.

High-resolution mass spectra of **1** was recorded by Dr. Javier Calvo on UltrafleXtreme III MALDI tandem mass spectrometer (Bruker) in reflector acquisition operation mode and the samples were prepared in chloroform.

ATR-FTIR spectra were recorded on a Bruker ALPHA ATR-IR spectrometer. TA Instruments Discovery system was used to perform the thermogravimetric analysis (TGA) using a 10 ºC min^{-1} heating rate under a nitrogen flow, which was changed to oxygen from 800 ºC.

Routine powder X-ray diffraction (PXRD) patterns for the optimization of the reaction conditions were collected by using a PHILIPS X'PERT PRO automatic diffractometer operating at 40 kV and 40 mA, in theta-theta configuration, secondary monochromator with Cu-Kα radiation (λ = 1.5418 Å) and a PIXcel solid state detector (active length in 2θ 3.347º). Data were collected from 1 to 50° 2θ (step size = 0.026 and time per step = 300 s, total time 40 min) at room temperature. A variable divergence slit, giving a constant 4.0 mm area of sample illumination, was used.

PXRD patterns for Pawley refinements were collected in a Panalytical Empyrean X'pert Pro diffractometer operating at 45kV and 40 mA with a Debye-Scherrer geometry and Cu-Kα radiation (λ = 1.5418 Å) and a PIXcel detector. Profiles were collected at room temperature using Soller Slits of 0.02° and a divergence slit of ¼ in the angular range of $2° < 2\theta < 90°$ with a step size of 0.013°. Pawley refinements were carried out with Topas Academic v6 (http://www.topas-academic.net/). The background was fitted with an 18-coefficients Chebyshev polynomial functions and peak shapes were modelled with a Thompson-Cox-Hastings pseudo-Voigt profile function. The parameters of the final Pawley refinement are summarised in Table S2.

Transmission electron microscopy (TEM) was performed using a JEOL 2100PLUS transmission electron microscope with an accelerating voltage of 80 kV or 200 kV and an Gatan UltraScan 1000XP camera, located at the University of Nottingham Nanoscale and Microscale Research Centre. Samples were prepared by dispersing the dry sample in HPLC grade IPA and drop-casting onto a copper TEM grid coated with "lacey" carbon film (Agar Scientific UK) using a Pasteur pipette. Analysis was performed using Gatan Microscopy Suite 3.21 and ImageJ FIJI software. Scanning electron microscopy (SEM) images were acquired using a Zeiss Crossbeam 550 FIBSEM at an accelerating voltage of 2 kV and current of 80 pA or 100 pA at a working distance of 4.0 mm on a lacy carbon coated TEM grid. Analysis was performed using SmartTiff (v03.00.03) and ImageJ FIJI software.

MicroED Structure determination. For MicroED analysis, the powdered sample was added to lacey carbon EM grids. Excess sample was removed by gently tapping the tweezers holding the EM grids. Electron diffraction data were collected using standard small molecule MicroED procedures[3-5]. The samples were loaded into a Titan Krios cryo-TEM operated at 300 kV and at cryogenic temperature. The cryo-TEM is equipped with a CETA D detector which was used for identifying crystals, collecting initial diffraction data, and collecting MicroED data sets, and all of these steps were performed with the cryo-TEM in "low dose" mode. Crystals were located on the grid using a low magnification search (LM mode, ~600x) and the quality of the nanocrystals was determined by collecting initial selected area diffraction patterns. Full continuous rotation MicroED data sets were then collected from those crystals that showed high quality diffraction in the initial screening. MicroED data sets were indexed, integrated, and scaled using XDS[6]. Ultimately the structure of **Bet-P-1** was determined by merging MicroED data from 4 crystals, and the structure was solved by direct methods in SHELXT[7] and refinement using SHELXL[8]. The data collection, processing, and refinement statistics can be found in Table S1.

The porosity of the structure was evaluated by nitrogen sorption isotherms, measured at 77 K with a Micromeritics 3Flex apparatus. The sample was degassed in an Autosorb station at and 10^{-6} Torr at 100 °C prior to analysis. Surface area and volume values were calculated from nitrogen adsorption-desorption isotherms (77 K). Specific surface area (SA) was calculated by multi-point Brunauer-Emmett-Teller (BET) method.

The surface areas were computed with Poreblazer 4.0[9] and Zeo++,[10, 11] for Poreblazer the default force-field and coefficients were used with a cubelet size of 0.2 Å while for Zeo++ a probe radius of 1.86 Å were applied.

Solid-state UV-Vis-NIR electronic absorption spectra were recorded on a PerkinElmer Lambda 950 UV/VIS/NIR spectrophotometer by solid-state electronic diffuse reflectance.

Hydrolysis/digestion of **Bet-P-1**. 1 mg of **Bet-P-1** was dispersed in 0.5 mL of deuterated trifluoroacetic acid (TFA-d_1) in a vial. The dispersion was stirred at room temperature until the polymer was completely dissolved (approximately 48 h).

Computer models of were performed at the Density Functional Theory (DFT) level starting from the ED derived crystalline structure of **Bet-P-1** with the Fritz Haber Institute ab initio molecular simulations (FHI-aims) package.[12-14] For this, "light" numeric atomic orbitals, which approximately correspond to TZVP level of calculations were used with the PBE functional augmented with van der Waals (Tkatchenko-Scheffler) dispersion correction unless otherwise stated. Band structure and band gap calculations were performed on the crystalline experimental structure after partial optimization of the hydrogen atoms with an 8x4x2 grid of k-points. Frontier orbitals were rendered in real space, from Γ-point calculations on a 2x2x2 supercell.

FP-TRMC experiments were conducted for the sample on a quartz plate using the third harmonic generator (THG; 355 nm) of a Nd:YAG laser (Continuum Inc., Surelite II, 5–8 ns pulse duration, 10 Hz) as the excitation source (9.1×10^{15} photons cm^{-2} $pulse^{-1}$). The frequency and power of microwave were ~9.1 GHz and 3 mW, respectively. The photoconductivity transient $\Delta\sigma$ was converted to the product of the quantum yield (φ) and the sum of charge carrier mobilities $\Sigma\mu$ ($= \mu_+ + \mu_-$) by the formula $\varphi\Sigma\mu = \Delta\sigma(eI_0F_{light})^{-1}$, where e and F_{light} are the unit charge of a single electron and a correction (or filling) factor, respectively.

Synthesis of tetrabromodibenzotetraazahexacene, 5

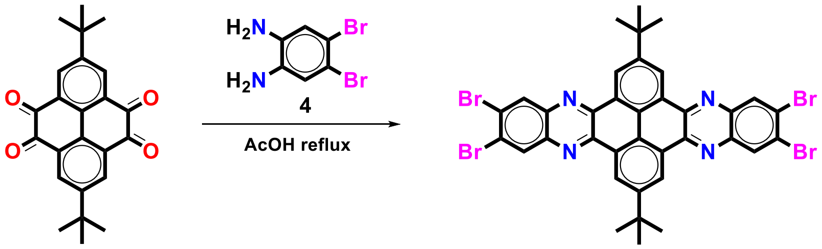

3 5

Compound **3** (0.80 g, 2.14 mmol) and compound **4** (1.19 g, 4.49 mmol) were dissolved in acetic acid (100 mL) and the suspension was stirred at reflux temperature 48 h. After cooling to room temperature the mixture was poured into ice water and the precipitate was collected by suction filtration. The precipitate was triturated in methanol and filtered off, and the solid was washed several times with methanol, ethyl acetate and dichloromethane to afford **5** as a yellow powder (1.68 g, 95 %).

^{1}H NMR (400 MHz, TFA-d_1): δ 10.05 (s, 4H), 9.13 (s, 4H), 1.83 (s, 18H).

^{13}C NMR: Compound **5** is not sufficiently soluble for recording a ^{13}C NMR spectrum.

MS (MALDI-TOF): [M+H]$^+$ Calculated for $C_{36}H_{26}Br_4N_4$, m/z 834.8879; Experimental m/z 834.8909.

Synthesis of dibenzotetraazahexacene, 1

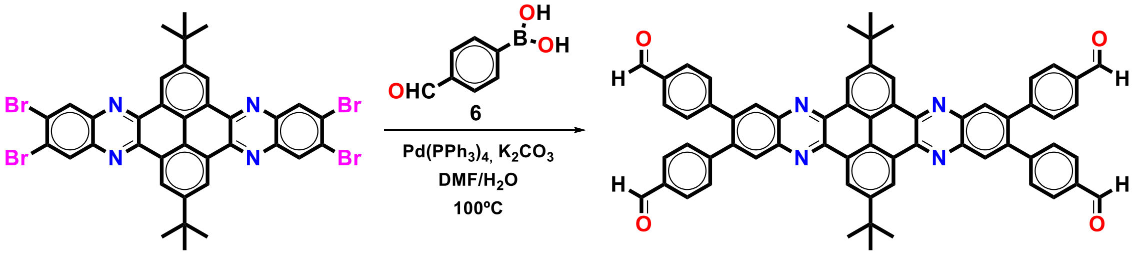

5 1

Compound **5** (0.25 g, 0.30 mmol), compound **6** (0.36 g, 2.41 mmol), $Pd(PPh_3)_4$ (0.07 g, 0.06 mmol) and K_2CO_3 (0.13 g, 0.96 mmol) were added to a 100 mL round bottom flask and degassed by pump-thaw cycles. Separately, DMF (13 mL) and water (4 mL) were degassed by bubbling nitrogen for 15 min. Then, the degassed solvents were added to the reaction mixture under nitrogen and heated at 100 °C for 72 h. The organic phase was dried over sodium sulfate and the solvent was evaporated. Crude product was triturated in methanol, filtered, and washed several times with methanol and ethyl acetate. The residue

was then subjected to chromatography (chloroform) to afford **1** as a yellow powder (63 mg, 22 %).

^{1}H NMR (400 MHz, $CDCl_3$): δ 10.07 (s, 4H), 9.85 (s, 4H), 8.61 (s, 4H), 7.89-7.87 (d, 8H), 7.56-7.54 (d, 8H), 1.76 (s, 18H).

^{13}C NMR (100 MHz, 1,1,2,2-tetrachloroethane-d_2): δ 191.32, 151.38, 146.09, 143.94, 141.51, 141.45, 135.38, 131.03, 130.48, 129.33, 129.19, 125.93, 125.28, 120.20, 35.72, 31.65.

MS (MALDI-TOF) (m/z): $[M+H]^+$ Calculated for $C_{64}H_{46}N_4O_4$ 935.3590; found 935.3534.

Synthesis of Bet-P-1

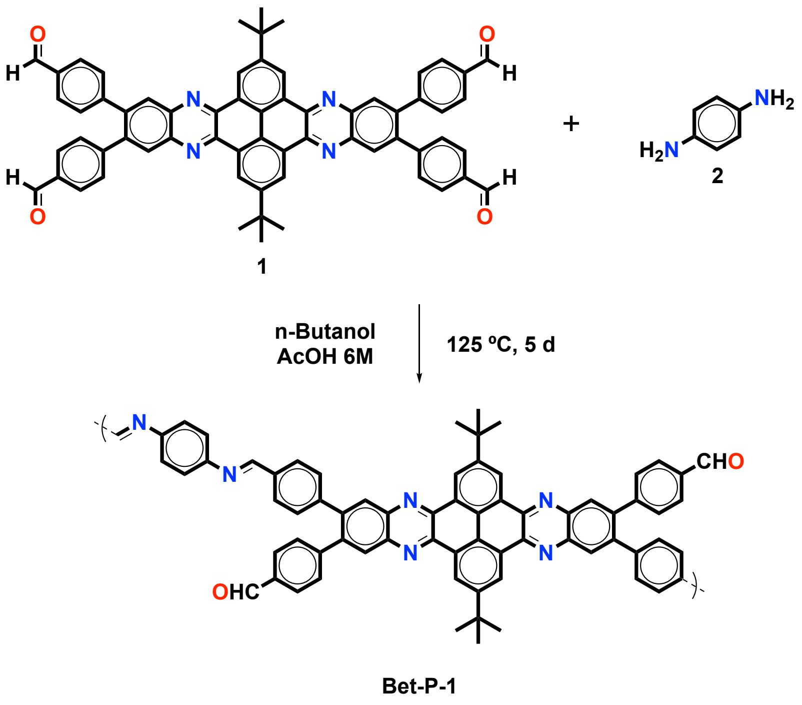

Compound **1** (6 mg, 6.4 μmol) and compound **2** (1.39 mg, 12.8 μmol) were sonicated in 0.5 mL of n-butanol in a pre-scored 2 mL ampoule. Then, acetic acid 6M (aq.) (0.05 mL) was added to the mixture. The suspension was degassed by using three freeze-pump-thaw cycles. The ampoule was sealed off using flame and heated at 125 °C for 5 d. The yellow precipitate was collected by filtration and washed five times with anhydrous tetrahydrofuran, chloroform, and acetone. The powder was dried at 85 °C under vacuum for 12 h affording 6.81 mg of **Bet-P-1** as a yellow powder (90 % yield).

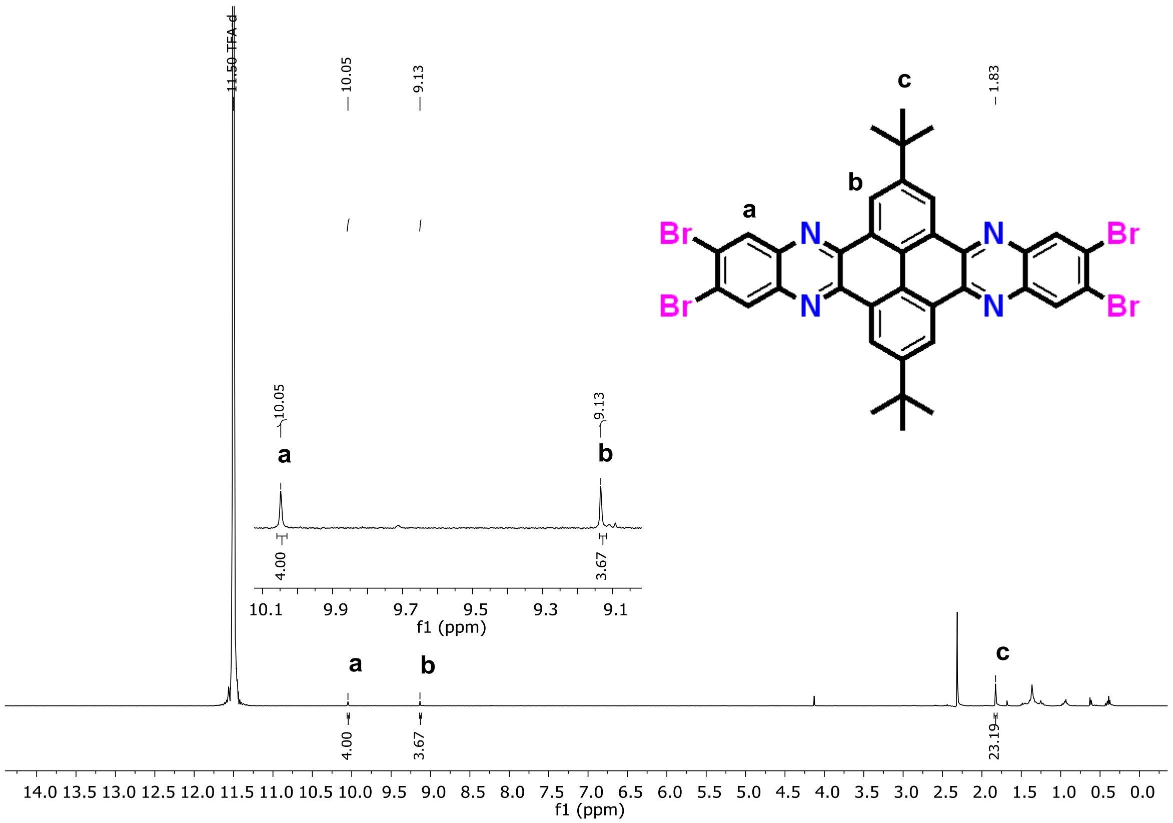

Figure S9. ^{1}H NMR spectrum of tetrabromodibenzotetraazahexacene, **5** (400 MHz, TFA-d_1, 298 K).

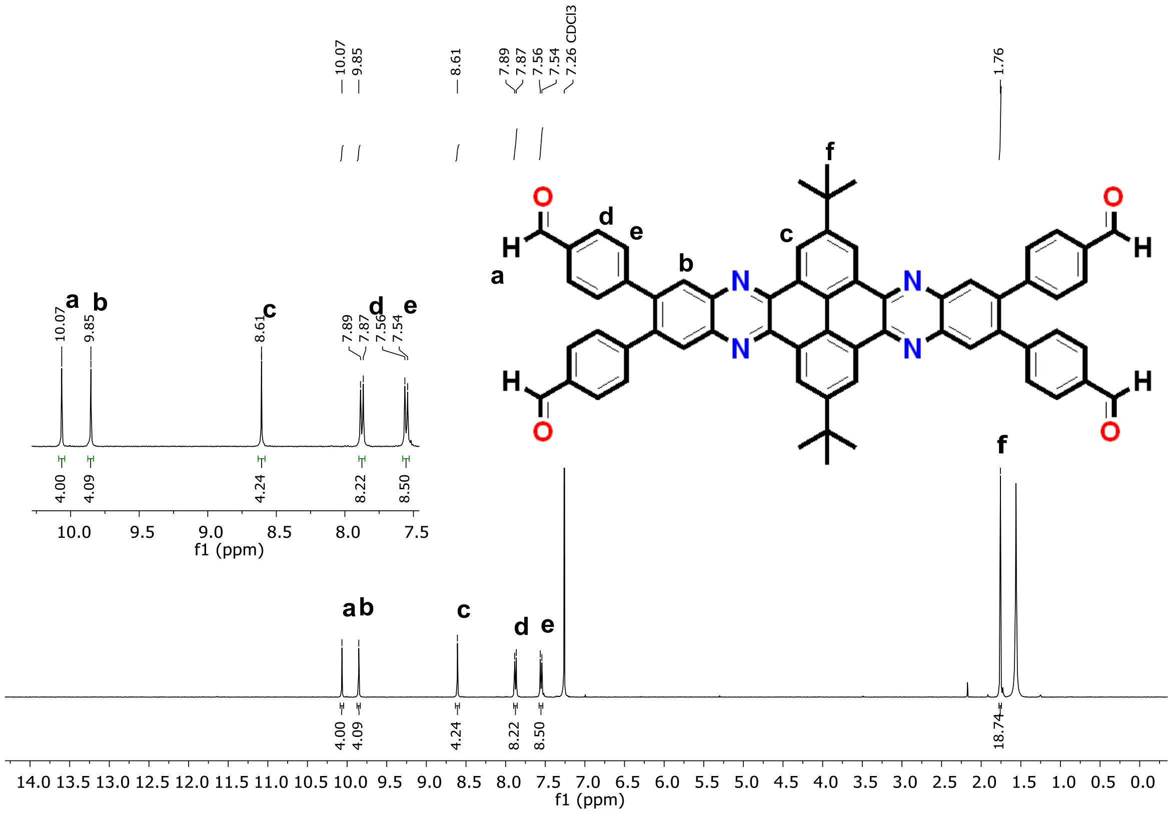

Figure S10. ^{1}H NMR spectrum of dibenzotetraazahexacene, **1** (400 MHz, $CDCl_3$, 298 K).

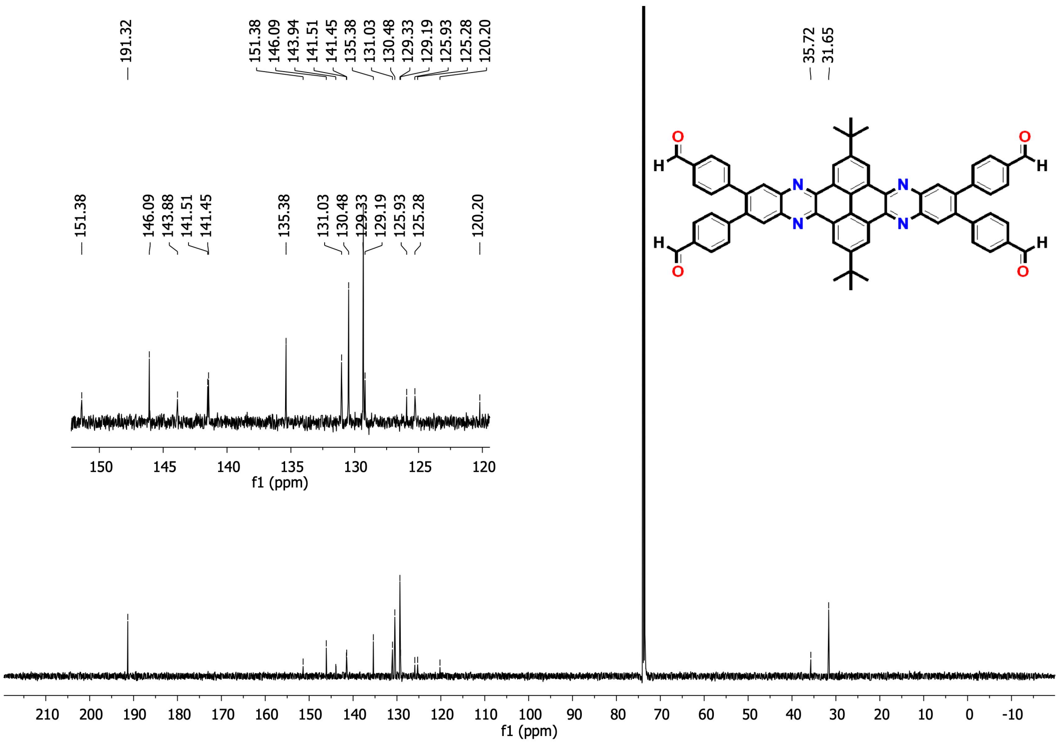

Figure S11. ^{13}C NMR spectrum of dibenzotetraazahexacene **1** (100 MHz, 1,1,2,2-tetrachloroethane-d_2, 298 K).

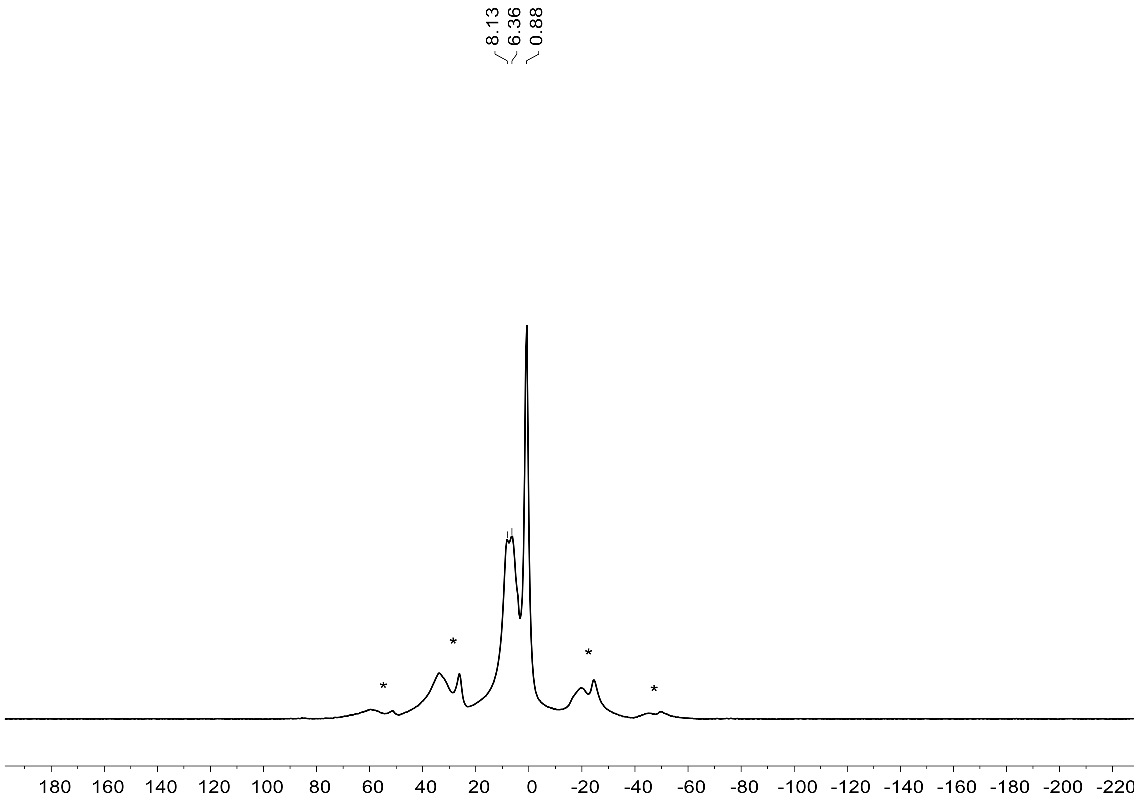

Figure S12. Solid-state ^{1}H NMR spectrum of **Bet-P-1** (* indicate spinning side bands).

References

1. Yamato, T.; Fujimoto, M.; Miyazawa, A.; Matsuo, K., Selective preparation of polycyclic aromatic hydrocarbons. Part 5.1 Bromination of 2,7-di-tert-butylpyrene and conversion into pyrenoquinones and their pyrenoquinhydrones. *J. Chem. Soc., Perkin Trans. 1* **1997,** (8), 1201-1208.
2. El-Assaad, T. H.; Parida, K. N.; Cesario, M. F.; McGrath, D. V., Sterically driven metal-free oxidation of 2,7-di-tert-butylpyrene. *Green Chem.* **2020,** *22* (18), 5966-5971.
3. Nannenga, B. L.; Gonen, T., The cryo-EM method microcrystal electron diffraction (MicroED). *Nat. Methods* **2019,** *16* (5), 369-379.
4. Levine, A. M.; Bu, G.; Biswas, S.; Tsai, E. H. R.; Braunschweig, A. B.; Nannenga, B. L., Crystal structure and orientation of organic semiconductor thin films by microcrystal electron diffraction and grazing-incidence wide-angle X-ray scattering. *Chem. Commun.* **2020,** *56* (30), 4204-4207.
5. Levine, A. M.; He, G.; Bu, G.; Ramos, P.; Wu, F.; Soliman, A.; Serrano, J.; Pietraru, D.; Chan, C.; Batteas, J. D.; Kowalczyk, M.; Jang, S. J.; Nannenga, B. L.; Sfeir, M. Y.; R. Tsai, E. H.; Braunschweig, A. B., Efficient Free Triplet Generation Follows Singlet Fission in Diketopyrrolopyrrole Polymorphs with Goldilocks Coupling. *J. Phys. Chem. C* **2021,** *125* (22), 12207-12213.
6. Kabsch, W., Xds. *Acta Crystallogr. D* **2010,** *66* (Pt 2), 125-32.
7. Sheldrick, G. M., SHELXT - integrated space-group and crystal-structure determination. *Acta Crystallogr. A* **2015,** *71* (Pt 1), 3-8.
8. Sheldrick, G. M., Crystal structure refinement with SHELXL. *Acta Crystallogr. C* **2015,** *71* (Pt 1), 3-8.
9. Sarkisov, L.; Bueno-Perez, R.; Sutharson, M.; Fairen-Jimenez, D., Materials Informatics with PoreBlazer v4.0 and the CSD MOF Database. *Chem. Mater.* **2020,** *32* (23), 9849-9867.
10. Willems, T. F.; Rycroft, C. H.; Kazi, M.; Meza, J. C.; Haranczyk, M., Algorithms and tools for high-throughput geometry-based analysis of crystalline porous materials. *Micropor. Mesopor. Mat.* **2012,** *149* (1), 134-141.
11. Martin, R. L.; Haranczyk, M., Construction and Characterization of Structure Models of Crystalline Porous Polymers. *Cryst. Growth Des.* **2014,** *14* (5), 2431-2440.
12. Blum, V.; Gehrke, R.; Hanke, F.; Havu, P.; Havu, V.; Ren, X.; Reuter, K.; Scheffler, M., Ab initio molecular simulations with numeric atom-centered orbitals. *Comput. Phys. Commun.* **2009,** *180* (11), 2175-2196.
13. Marek, A.; Blum, V.; Johanni, R.; Havu, V.; Lang, B.; Auckenthaler, T.; Heinecke, A.; Bungartz, H. J.; Lederer, H., The ELPA library: scalable parallel eigenvalue solutions for electronic structure theory and computational science. *J. Phys. Condens. Matter* **2014,** *26* (21), 213201.
14. Yu, V. W.-z.; Corsetti, F.; García, A.; Huhn, W. P.; Jacquelin, M.; Jia, W.; Lange, B.; Lin, L.; Lu, J.; Mi, W.; Seifitokaldani, A.; Vázquez-Mayagoitia, Á.; Yang, C.; Yang, H.; Blum, V., ELSI: A unified software interface for Kohn–Sham electronic structure solvers. *Comput. Phys. Commun.* **2018,** *222*, 267-285.
